# Immunohistochemical Analysis of Cyclooxygenase-2 in Non-Melanocytic Skin Cancer: Correlation With Morphological Subtype and Histologic Grade

**DOI:** 10.14740/wjon869w

**Published:** 2014-12-03

**Authors:** Ali Koyuncuer

**Affiliations:** Department of Pathology, Antakya State Hospital, Hatay, Turkey. Email: alikoyuncuer@hotmail.com

**Keywords:** Cancer, COX-2, Non-melanoma, Skin

## Abstract

**Background:**

Basal cell carcinomas (BCCs) and squamous cell carcinomas (SCCs) are known as non-melanoma skin cancers (NMSCs), and they account for approximately 90% of all skin cancers. Cyclooxygenase-2 (COX-2) is expressed in NMSC and in premalignant cutaneous lesions (actinic keratosis).

**Methods:**

Immunohistochemistry was performed with COX-2 antibodies in skin biopsies (paraffin tissue archival blocks) from 28 cases with SCC and 33 cases with BCC.

**Results:**

COX-2 was immunostained in a total of 61 cases. There was no staining or weakly positive staining in 73.8% of the cases (45 cases), and there was moderate or strong positive staining in 26.3% of the cases (16 cases). COX-2 was expressed in 42.4% of the BCC cases and in 57.1% of the SCC cases. There was a significant relationship between positive COX-2 staining and SCC (P = 0.016).

**Conclusions:**

In this study, SCCs were significantly correlated with the expression of COX-2. In addition, COX-2 was more frequently expressed in SCC than in BCC. The largest diameters of the SCC were significantly correlated with the expression of COX-2. There were no significant associations between COX-2 staining and clinicopathologic features such as the ulceration of the tumor, its anatomic localization, patient gender, the histologic grade of the SCC and the morphological subtype of the BCC.

## Introduction

Non-melanoma skin cancers (NMSCs) are characterized by the proliferation of epidermal keratinocytes [1]. Basal cell carcinomas (BCCs) and squamous cell carcinomas (SCCs) are the most common types of cancer [2]. BCC is the most frequently diagnosed cancer in the United States, and has an incidence of approximately 0.5%. Follicular germinative cells may cause BCCs [3]. SCCs are malignant tumors of epidermal keratinocytes and occur in approximately 20% of all skin malignancies [4]. Actinic keratoses (AKs) occur on sun-exposed skin [5] and are known as “dysplastic keratinocytic epidermal lesions” [3]. The prevalence of AKs differs from country to country [6]. Frost et al reported that the prevalence of AKs in Australia ranged 40-60% in patients 40 years and older [7] and was 15% for men and 6% for women in England. Untreated lesions pose a risk for malignant transformation [6].

Cyclooxygenase (COX-1, COX-2) is an enzyme required for the conversion of arachidonic acid to prostaglandins (also known as prostanoids). Normally, COX-2 expression is not detectable or is very low, but it generally increases with pain and inflammation [8]. COX-2 protein was detected in the perinuclear region of normal cells [9] and in the cytoplasm of tumor cells, particularly in the perinuclear membranes of keratinocytes [10]. In some studies, non-steroidal anti-inflammatory drugs (NSAIDs) have been reported to inhibit carcinogenesis in humans and rodents. COX-2 is the isoform that has been implicated in the development of tumors [11]. The overexpression of COX-2 has been reported to cause different neoplasms, gastrointestinal cancers, breast tumors [12] and skin tumors [13]. The p53 activates proteins that are important in the process of DNA repair. If p53 does not activate these proteins, apoptosis occurs [14]. A mutation in p53 was observed in 40-50% of all skin cancers in normal people [15]. COX-2 inhibitors (NSAID) are effective chemopreventive agents in NMSC. Other useful agents include retinoids and oral difluoromethylornithine [16].

## Materials and Methods

### Immunohistochemical analysis

A total of 61 paraffin tissue archival blocks (4 μm thick, 10% neutral buffered formalin fixation) were immunohistochemically stained with COX-2 (1:100 diluted rabbit polyclonal antibody, Thermo Scientific). Primary antibody labeling was performed with an automated Ventana Ultraview DAB detection kit in a BenchMark XT (Ventana Medical Systems, Inc.). The study included 61 randomly selected incident cases with newly diagnosed NMSC, which was histologically confirmed. A minimum of 150 - 200 tumor cells were scored per patient (using × 40 objective). COX-2 immunohistochemical staining was considered positive if it was cytoplasmic or perinuclear.

The intensity of staining was scored as 0, 1, 2, or 3 as follows. 1) Score 0: no staining; 2) Score 1: weak/diffuse cytoplasmic staining (< 10% stained cells); 3) Score 2: moderate staining (10-90% stained cells); 4) Score 3: strong/intensive staining (> 90% stained cells). Low-grade adenocarcinoma of the colon was used as a positive external control for COX-2 staining.

### Statistical analyses

Data were analyzed using Statistical Package for Social Sciences (SPSS) software (version 21.0 for Windows). A *t*-test was used to compare averages, a Ki-test was used to determine the dispersion of the two groups (BCC vs. SCC), and a Kruskal-Wallis-H test was used to determine the dispersion of BCC group and SCC group. All differences associated with a chance probability of 0.05 or less were considered statistically significant. Continuous variables are presented as mean ± standard deviation (SD).

## Results

A total of 61 NMSC cases were examined. In this retrospective study, all skin specimens were evaluated. (Supplementary 1, www.wjon.org).

### Age

The overall mean age of the patients was 70.1 ± 14.87 years (range 29 - 98). The mean age of those diagnosed with BCC was 64.8 years, and the mean age of those diagnosed with SCC was 76.4 years.

### Gender

BCC was observed in 21 of the 33 men (54.1%), and in 12 of the 28 women (45.9%). SCCs were detected in 12 men and 16 women.

### Cancer type

BCC was observed in 33 (54.1%) patients, while SCC was observed in 28 patients (45.9%).

### Greatest tumor diameter

The mean diameter of all types of solitary tumors was 8.68 ± 4.88 mm. The mean tumor diameters for BCC and SCC were 6.87 ± 2.71 mm (range 3 - 12), and 10.8 ± 5.95 mm (range 3 - 23), respectively.

The greatest tumor diameter was ≤ 10 mm in 72.1% of the patients and was ≥ 11 mm in 27.9%. The greatest tumor diameter was ≤ 10 mm in 50% of those with SCC and in 90.9% of those with BCC, while the greatest tumor diameter was ≥ 11 mm in 50% of those with SCC and in 9.1% of those with BCC.

### BCC morphological subtype

Nodular (solid) subtype focal cystic changes were observed in 26 (42.7%) patients, mixed patterns were observed in three (4.8%), infiltrating type tumors were observed in one (1.6%), adenoid tumors were observed in one (1.6%), micronodular tumors were observed in one (1.6%), and basosquamous tumors were observed in one (1.6%) patient.

### SCC differentiation

Well-differentiated (grade 1) SCCs were detected in 15 cases (24.6%), while moderately differentiated (grade 2) SCCs were found in 13 cases (21.3%). We did not detect any poorly differentiated (grade 3) or anaplastic or undifferentiated (grade 4) tumors. This is most likely because we were able to make early diagnoses since the lesions developed on visible areas of the body.

### Anatomic localization

**Figure 1:**
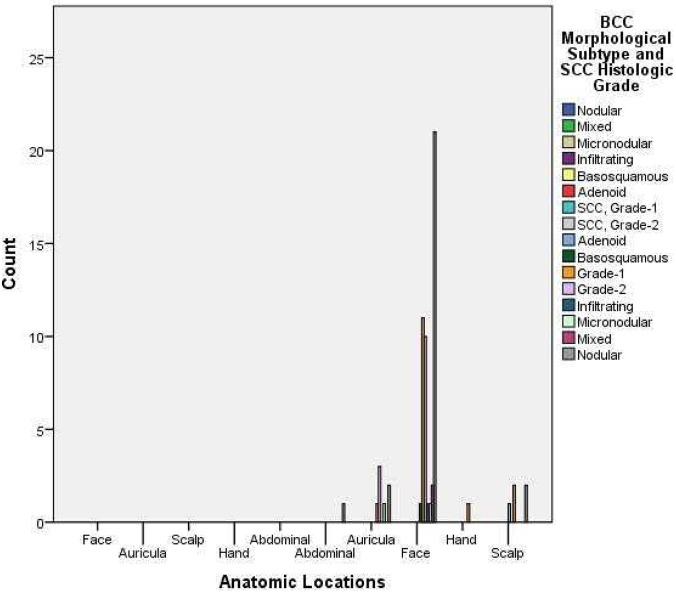
Distribution of BCC morphological subtype and SCC histologic grade.

All of the tumors were located on the face (77.1%), scalp (8.2%), and ear (11.5%). The tumors were located on the face of 78.8% of the BCC patients and on 75% of the SCC patients (Fig. 1).

### Ulceration

Ulceration was observed in 50.8% of all tumors. Ulceration was detected in 17 cases with BCC and in 14 cases with SCC.

### COX-2 expression and immunohistochemical analysis

**Figure 2:**
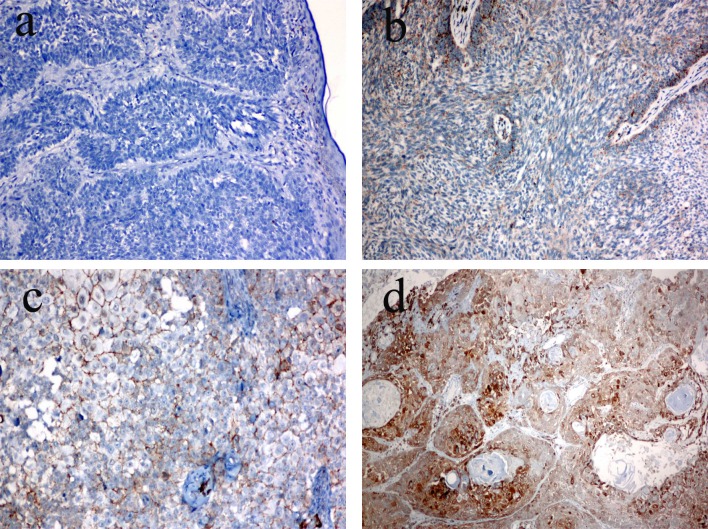
COX-2 expression in skin cancer. (a) Score 0 (no staining), BCC (× 20 objective). (b) Score 1 (weakly stained cells < 10%), BCC (× 10 objective). (c) Score 2 (moderately stained cells 10-90%), SCC (× 4 objective). (d) Score 3 (strong staining, > 90%), SCC (× 10 objective).

The expression of COX-2 in all cases was as follows: 31 cases (50.8%) had no COX-2 staining (score 0), 14 cases (23%) had < 10% positive cells (score 1), 12 cases (19.7%) had 10-90% positive cells (score 2), and four cases (6.6%) had > 90% positive cells (score 3) (Table 1). We stained for COX-2 in a total of 61 cases, and there was no staining or weakly positive staining in 45 cases (73.8%) and moderate or strong positive staining in 16 cases (26.3%) (Fig. 2). We observed no staining or weakly positive staining in 27 cases with BCC (81.8%) and moderately positive staining in six cases with BCC (18.2%), indicating that the majority of BCC cases had no or weak staining. There was no COX-2 immunoreactivity in 57.6% of the BCC cases. The cytoplasmic expression of COX-2 was moderately positive in five of the 26 nodular subtype BCC cases. No strongly positive COX-2 immunostaining was observed in any of the 33 cases with BCC. No statistically significant correlation was found between COX-2 expression and BCC morphological subtype (P = 0.297, P > 0.05). We observed no immunostaining or weakly positive staining in 18 SCC cases (64.3%) and moderately/strong positive staining in 10 SCC cases (35.7%). Results indicated that COX-2 staining was moderately/strong positive in 46.1% of the cases with moderately differentiated SCC. Strong and weak COX-2 cytoplasmic staining was observed in six cases with moderately differentiated (grade 2) SCC. COX-2 was more frequently expressed in SCC than in BCC. The histologic grade of the SCC was not significantly correlated to the expression of COX-2 (P = 0.494, P > 0.05).

**Table 1:** Distribution of COX-2 in Different Types of Skin Cancers

Cancer type	COX-2 score				
	0, % (n)	1, % (n)	2, % (n)	3, % (n)	Total, % (n)
BCC	31.15 (19)	13.11 (8)	9.83 (6)	0 (0)	54.09 (33)
SCC	19.7 (12)	9.83 (6)	9.83 (6)	6.55 (4)	45.91 (28)
Total, % (n)	50.85 (31)	22.94 (14)	19.66 (12)	6.55 (4)	100 (61)

COX-2 expression in tumors ≥ 11 mm in diameter was detected in three cases of BCC and in 10 cases of SCC. A significant relationship was found between positive COX-2 staining and SCC tumor size (P = 0.016, P < 0.05). However, no statistically significant difference was found between COX-2 expression and BCC tumor size (P = 0.495, P > 0.05). COX-2 expression of the tumor ulceration was observed in 41% of the BCC cases and in 50% of the SCC cases. The ulceration of the tumor was not significantly correlated to the expression of COX-2 (BCC: P = 0.763 and SCC: P = 0.510, P > 0.05). No statistically significant difference was found between COX-2 expression in BCC or SCC and anatomic localization (P = 0.939, P = 0.867, respectively). COX-2 staining was positive in 75% of the SCC patients with tumors localized on the face. No association was found between the COX-2 expression and gender (P = 0.903, P > 0.05). Although not significant, more women (16/28) than men (14/33) had elevated COX-2 expression.

## Discussion

MSCs and NMSCs are the most frequently seen types of cancer in Caucasians [17]. Jemal et al reported that there were more than 2,000,000 unreported cases of BCC and SCC in the USA in 2010 [18]. The incidence rate of NMSC is rising due to compound factors, including a reduced efficacy of the immune system, a decrease in the ozone layer, more time spent outdoors (sports, activities, jobs), and in some cases, heredity [17]. NMSCs are caused by environmental, immunologic and genetic factors [19]. COX is an enzyme involved in the conversion of arachidonic acid to prostanoids [12]. COX-2 is a tumor initiator that was first described by Kujubu et al in 1991 [20]. There have been contradictory reports on the expression of COX-2 in epithelial tumors and on normal skin. Others have reported that COX-2 expression is limited to keratinocytes (granular, spinous layer) of the cutaneous tissue [21]. However, Nijsten et al reported that COX-2 protein expression is absent (0%) in the normal epidermis [22]. In our study, the COX-2 expression pattern is more heterogeneous and diffuse in carcinomatous matter than in normal skin.

COX-2 expression is elevated in tumors located in the colon, stomach, esophagus, breast and lung [23]. COX-2 is strongly expressed in benign polyps (50%) and carcinomas of the colon (80-85%) [24]. COX-2 stimulates cancer cell to proliferation by protecting the tumor cells and by promoting angiogenesis [25]. Angiogenesis is stimulated by COX-2, transforming growth factor-β, and vascular endothelial growth factor in colorectal carcinoma [26]. In contrast, another study reported that the immunopositivity of COX-2 was not associated with cell proliferation or the grade of malignancy [27].

Mouse models have shown that the expression of COX-2 plays a role in AKs, SCC and BCC. COX-2 expression initiates significant responses in SCC and BCC in both mice and humans [28]. Tiano et al reported that many different factors may contribute to rat skin papilloma and tumorigenesis [11]. These studies indicate that COX-2 plays a significant role in cell proliferation, apoptosis, angiogenesis and cell invasiveness [29]. It has been reported that there is a significant correlation between the elevation of COX-2 and a decrease in survival of carcinoma of the cervix. In this study, there was a correlation between COX-2 expression and tumor size [30]. In our study, COX-2 expression was observed in 43.3% of the tumors ≥ 11 mm diameter (13/17 cases). Another study reported a correlation between high expression levels of COX-2 and the invasion of muscularis propria and tumor progression in the urothelial carcinoma of the bladder. This study showed that COX-2 expression has little prognostic significance to the urothelial carcinoma of the bladder. However, COX-2 inhibitors are often utilized for chemoprophylaxis and treatment of bladder cancers [31]. COX-2 is not expressed in the normal intestine. However, colorectal carcinomas produce very high levels of COX-2 (detected in 85% of cases). COX-2 overexpression increases metastatic potential. Several studies have reported that the use of aspirin and NSAIDs leads to a 40-50% decline in the mortality of patients with colon tumors [32]. Another study investigated COX-2 expression in gastric adenocarcinoma [33] and ovarian adenocarcinomas, and found that COX-2 expression in ovarian tumor cells is partially caused by the failure of p53 [34]. Invasive ductal carcinoma and ductal carcinoma *in situ* in breast cancers correlate with COX-2 expression in 85% and 74.5% of cases, respectively. Despite these findings, a recent report found no correlation between COX-2 expression and histological grade or tumor size [35].

The expression of COX-2 was reported to be weakly positive in 70% of cases (total of 22 cases) with Merkel cell carcinoma (primary cutaneous). The expression of COX-2 did not correlate with prognostic factors [36]. Several studies have investigated the expression of COX-2 as a way to detect cutaneous melanomas [37]. This marker has a particularly high level of sensitivity and specificity, and is advantageous in the differential diagnosis of early melanomas and benign melanocytic lesions [38]. Kim et al observed COX-2 expression in 50% of SCCs and in 80% of BCC cases, but detected no correlation between the COX-2 and p53 marker and skin tumors [13]. Amirnia et al detected COX-2 expression in 94% of non-melanocytic skin cancer of SCC and in 87.5% of that of BCC. COX-2 expression was detected in the malignant and premalignant epidermal lesions. It has been proposed that COX-2 expression may lead to the development of treatments for cutaneous tumors [23]. We found COX-2 protein expression in 57.1% of SCC cases and in 42.4% of BCC cases. In another study, the increased expression of COX-2 was detected in ultraviolet radiation-induced NMSCs [39]. Athar et al recommended a specific treatment regimen of COX-2 inhibitors for ultraviolet radiation (UVB)-induced cutaneous cancer [40]. Immunohistochemical studies have shown that the initiation of COX-2 expression and angiogenesis may play a role in the development of SCC [22].

Butler et al reported a relationship between the use of NSAIDs and a reduced risk of skin SCCs and AKs [41]. Elmets et al reported that chemoprophylaxis agents and NSAIDs (particularly the selective COX-2 inhibitors) can be effective in improving patients’ risk of developing skin cancers [16]. Fischer et al also reported that selective COX-2 inhibitors (NSAIDs) can be used to prevent skin cancers [42]. Nijsten et al reported that COX-2 was expressed in 31% of cases with AK, 22% of cases with Bowen’s disease, and in 40% of cases with SCC [22].

### Conclusions

The precise pathogenesis of skin cancer is difficult to associate with COX-2 expression. Further studies are needed to explain the role of COX-2 in skin cancers. Nevertheless, we observed a correlation between increases in COX-2 immunoreactivity and skin cancer. In particular, COX-2 protein expression was increased in SCC. We also found that COX-2 expression was positively correlated with tumor size. Immunohistochemical results showed that COX-2 has a heterogeneous distribution in skin cancer cells. NSAIDs have preventative effects (molecular target) on skin cancers, which may be in part responsible for favorable results in the long-term survival of patients and for the inhibition of tumor growth, invasion and metastasis.

## Abbreviations

BCCs: basal cell carcinomas
SCCs: squamous cell carcinomas
AKs: actinic keratoses
COX: cyclooxygenase
NSAID: non-steroidal anti-inflammatory drug

## References

[1] Weedon D, Marks R, Kao GF, Harword CA, LeBoit PE, Burg G, Weedon D, Sarasin A. Lyon (2006). Keratinocytic Tumours. World Health Organization Classification of Tumours Pathology & Genetics of Skin Tumours.

[2] Ramos J, Villa J, Ruiz A, Armstrong R, Matta J (2004). UV dose determines key characteristics of nonmelanoma skin cancer. Cancer Epidemiol Biomarkers Prev.

[3] Patel RV, Frankel A, Goldenberg G (2011). An update on nonmelanoma skin cancer. J Clin Aesthet Dermatol.

[4] Correa LH, Popoaski CP, Custodio G, Goncalves CO, Trevisol FS (2012). Epidemiology of squamous cell carcinomas among the population attended in the city of Tubarao, Brazil, between 1999 and 2009. An Bras Dermatol.

[5] Goldberg LH, Mamelak AJ (2010). Review of actinic keratosis. Part I: etiology, epidemiology and clinical presentation. J Drugs Dermatol.

[6] Weedon D, Weedon D (2010). Tumors of the epidermis. Weedon's Skin Pathology.

[7] Frost CA, Green AC (1994). Epidemiology of solar keratoses. Br J Dermatol.

[8] Murphy HS, Rubin R, Strayer DS (2012). In?ammation. Rubin's Pathology. Clinicopathologic Foundations of Medicine.

[9] Steinhoff M, Groves RW, LeBoit PE, Luger TA, Burns T, Breathnach S, Cox N, Grif C (2010). In?ammation. Rook's Textbook of Dermatology.

[10] Furstenberger G, Marks F, Muller-Decker K (2003). Cyclooxygenase-2 and skin carcinogenesis. Prog Exp Tumor Res.

[11] Tiano HF, Loftin CD, Akunda J, Lee CA, Spalding J, Sessoms A, Dunson DB (2002). Deficiency of either cyclooxygenase (COX)-1 or COX-2 alters epidermal differentiation and reduces mouse skin tumorigenesis. Cancer Res.

[12] Kagoura M, Toyoda M, Matsui C, Morohashi M (2001). Immunohistochemical expression of cyclooxygenase-2 in skin cancers. J Cutan Pathol.

[13] Kim KH, Park EJ, Seo YJ, Cho HS, Kim CW, Kim KJ, Park HR (2006). Immunohistochemical study of cyclooxygenase-2 and p53 expression in skin tumors. J Dermatol.

[14] Rubin R, Strayer DS, Rubin R, Strayer DS (2012). Cell Adaptation, Cell Injury and Cell Death. Rubin's Pathology Clinicopathologic Foundations of Medicine.

[15] Sarasin A, Giglia-Mari G (2002). p53 gene mutations in human skin cancers. Exp Dermatol.

[16] Elmets CA, Viner JL, Pentland AP, Cantrell W, Lin HY, Bailey H, Kang S (2010). Chemoprevention of nonmelanoma skin cancer with celecoxib: a randomized, double-blind, placebo-controlled trial. J Natl Cancer Inst.

[17] Leiter U, Garbe C (2008). Epidemiology of melanoma and nonmelanoma skin cancer—the role of sunlight. Adv Exp Med Biol.

[18] Jemal A, Siegel R, Xu J, Ward E (2010). Cancer statistics, 2010. CA Cancer J Clin.

[19] Loeb KR, Asgari MM, Hawes SE, Feng Q, Stern JE, Jiang M, Argenyi ZB (2012). Analysis of Tp53 codon 72 polymorphisms, Tp53 mutations, and HPV infection in cutaneous squamous cell carcinomas. PLoS One.

[20] Bakhle YS (2001). COX-2 and cancer: a new approach to an old problem. Br J Pharmacol.

[21] Kuzbicki L, Lange D, Stanek-Widera A, Chwirot BW (2011). Different expression of cyclooxygenase-2 (COX-2) in selected nonmelanocytic human cutaneous lesions. Folia Histochem Cytobiol.

[22] Nijsten T, Colpaert CG, Vermeulen PB, Harris AL, Van Marck E, Lambert J (2004). Cyclooxygenase-2 expression and angiogenesis in squamous cell carcinoma of the skin and its precursors: a paired immunohistochemical study of 35 cases. Br J Dermatol.

[23] Amirnia M, Babaie-Ghazani A, Fakhrjou A, Khodaeiani E, Alikhah H, Naghavi-behzad M, Zarrintan A (2014). Immunohistochemical study of cyclooxygenase-2 in skin tumors. J Dermatolog Treat.

[24] Williams CS, Mann M, DuBois RN (1999). The role of cyclooxygenases in inflammation, cancer, and development. Oncogene.

[25] Masferrer JL, Leahy KM, Koki AT, Zweifel BS, Settle SL, Woerner BM, Edwards DA (2000). Antiangiogenic and antitumor activities of cyclooxygenase-2 inhibitors. Cancer Res.

[26] Fosslien E (2001). Review: molecular pathology of cyclooxygenase-2 in cancer-induced angiogenesis. Ann Clin Lab Sci.

[27] Sakuma K, Fujimori T, Hirabayashi K, Terano A (1999). Cyclooxygenase (COX)-2 immunoreactivity and relationship to p53 and Ki-67 expression in colorectal cancer. J Gastroenterol.

[28] An KP, Athar M, Tang X, Katiyar SK, Russo J, Beech J, Aszterbaum M (2002). Cyclooxygenase-2 expression in murine and human nonmelanoma skin cancers: implications for therapeutic approaches. Photochem Photobiol.

[29] Fodera D, Lampiasi N, Cusimano A, Cervello M Cyclooxygenases in Cancer. In COX-2 Inhibitor Research. Edited by Maynard J. Howardell. New York: Nova Science Publishersi.

[30] Gaffney DK, Holden J, Davis M, Zempolich K, Murphy KJ, Dodson M (2001). Elevated cyclooxygenase-2 expression correlates with diminished survival in carcinoma of the cervix treated with radiotherapy. Int J Radiat Oncol Biol Phys.

[31] Shariat SF, Matsumoto K, Kim J, Ayala GE, Zhou JH, Jian W, Benedict WF (2003). Correlation of cyclooxygenase-2 expression with molecular markers, pathological features and clinical outcome of transitional cell carcinoma of the bladder. J Urol.

[32] Tsujii M, Kawano S, DuBois RN (1997). Cyclooxygenase-2 expression in human colon cancer cells increases metastatic potential. Proc Natl Acad Sci U S A.

[33] Ristimaki A, Honkanen N, Jankala H, Sipponen P, Harkonen M (1997). Expression of cyclooxygenase-2 in human gastric carcinoma. Cancer Res.

[34] Shigemasa K, Tian X, Gu L, Shiroyama Y, Nagai N, Ohama K (2003). Expression of cyclooxygenase-2 and its relationship to p53 accumulation in ovarian adenocarcinomas. Int J Oncol.

[35] Martins MM, de Oliveira VM, Lucarelli AP, M Silva MALG, Piato S, Francisco Rinaldi JF, Aoki JT (2008). Cyclooxygenase-2 and p53 Immunohistochemical expression in simultaneous ductal carcinoma in situ and invasive ductal carcinoma. App Cancer Res.

[36] Koljonen V, Lassus P, Tukiainen E, Ristimaki A, Haglund C, Bohling T (2005). Cyclooxygenase-2 expression in primary Merkel cell carcinoma. J Cutan Pathol.

[37] Kuzbicki L, Lange D, Chwirot BW (2009). Cyclooxygenase-2 immunohistochemistry in human melanoma: differences between results obtained with different antibodies. Melanoma Res.

[38] Chwirot BW, Kuzbicki L (2007). Cyclooxygenase-2 (COX-2): first immunohistochemical marker distinguishing early cutaneous melanomas from benign melanocytic skin tumours. Melanoma Res.

[39] Rundhaug JE, Mikulec C, Pavone A, Fischer SM (2007). A role for cyclooxygenase-2 in ultraviolet light-induced skin carcinogenesis. Mol Carcinog.

[40] Athar M, An KP, Morel KD, Kim AL, Aszterbaum M, Longley J, Epstein EH (2001). Ultraviolet B(UVB)-induced cox-2 expression in murine skin: an immunohistochemical study. Biochem Biophys Res Commun.

[41] Butler GJ, Neale R, Green AC, Pandeya N, Whiteman DC (2005). Nonsteroidal anti-inflammatory drugs and the risk of actinic keratoses and squamous cell cancers of the skin. J Am Acad Dermatol.

[42] Fischer SM, Lo HH, Gordon GB, Seibert K, Kelloff G, Lubet RA, Conti CJ (1999). Chemopreventive activity of celecoxib, a specific cyclooxygenase-2 inhibitor, and indomethacin against ultraviolet light-induced skin carcinogenesis. Mol Carcinog.

